# Pediatric anti-NMDA receptor encephalitis with catatonia: treatment with electroconvulsive therapy

**DOI:** 10.1186/s12969-019-0310-0

**Published:** 2019-02-18

**Authors:** Taha Moussa, Khalid Afzal, Joseph Cooper, Ryan Rosenberger, Karyn Gerstle, Linda Wagner-Weiner

**Affiliations:** 1 Section of Pediatric Rheumatology, Department of Pediatrics, University of Chicago Medical Center, 5841 S. Maryland Avenue, MC 5044, Chicago, 60637 IL USA; 2 Section of Pediatric Psychiatry, Department of Psychiatry, University of Chicago Medical Center, Chicago, USA; 3 Department of Psychiatry, University of Chicago Medical Center, Chicago, USA; 4 Department of Pediatrics, University of Chicago Medical Center, Chicago, USA

**Keywords:** Anti-NMDA receptor encephalitis, Catatonia, Electroconvulsive therapy, Plasma exchange, Rituximab, Intravenous immunoglobulins, Corticosteroids

## Abstract

**Background:**

Anti-NMDA receptor encephalitis, an autoimmune disease associated with antibodies against N-methyl-D-aspartate (NMDA) receptors, is being diagnosed more frequently, especially in children and young adults. Acute neurological and psychiatric manifestations are the common presenting symptoms. Diagnosing anti-NMDA receptor encephalitis is often challenging given the wide range of clinical presentation, and may be further complicated by its overlap of symptoms, brain MRI changes, and CSF findings with other entities affecting the brain. Even though diagnosis can be made by identifying antibodies in immune-mediated encephalitis, the diagnosis may be delayed by weeks to months. Delay in initiation of treatment with immune suppressive therapies is shown to be associated with adverse outcomes. Malignant catatonia is a severe and life-threatening state associated with anti-NMDA receptor encephalitis. It is often inadequately assessed and may not respond to immunosuppressive treatment.

**Case presentation:**

We present a confirmed case of anti-NMDA receptor encephalitis in a 16 year old girl who had severe critical neurological and psychiatric manifestations, including malignant catatonia and autonomic instability. Our patient continued to manifest malignant catatonia despite the initiation of prompt, aggressive immune suppressive therapies, including corticosteroids, plasmapheresis, intravenous gammaglobulin and rituximab, as well as treatment with high-dose benzodiazepines. Once electroconvulsive therapy (ECT) began, she had a robust response with resolution of her catatonia. Six weeks after treatment with eight ECT cycles, she had returned to her normal baseline cognitive and motor function.

**Conclusions:**

ECT was an effective and well-tolerated therapy in our patient, and should be considered for the treatment of children with anti-NMDA receptor encephalitis whose catatonia does not respond to immunosuppression and benzodiazepines.

## Introduction

In 2007, antibodies against N-methyl-D-aspartate (NMDA) receptor were identified in the hippocampus and forebrain of 12 cases of paraneoplastic encephalitis leading to the discovery of anti-NMDA receptor encephalitis [1]. Since then, the diagnosis of anti-NMDA receptor encephalitis is increasingly recognized, especially in children [2, 3]. A 2014 study found that 65% of anti-NMDA receptor encephalitis cases were in patients 18 years old or younger, and it is diagnosed four times more frequently than herpes simplex virus-1 (HSV-1), West Nile virus (WNV), or varicella-zoster virus (VZV) encephalitis in the same population [4]. The presenting phenotype may vary according to the age at onset: seizure, movement, and speech disorders ordinarily occur more often in younger children, while behavioral disorders, cognitive dysfunction, and memory deficits predominate in adolescents and adults [5].

Children and adults with encephalitis from a 2005–2006 cohort study were tested retrospectively for antibodies to NMDA receptor and voltage-gated potassium channel, highlighting the potential consequences related to untreated autoimmune encephalitis. Of the 16 patients who were positive for either of these antibodies, 38% had a severe disability, 38% had moderate disability, and only one patient had a good outcome [3]. Although there is limited evidence of the long-term effectiveness of current treatment modalities, first-line immune suppressive therapy with high dose corticosteroids combined with one, or both, intravenous immunoglobulin (IVIG) and plasma exchange (PLEX) demonstrates some improvement after 4 weeks. However, about one-half of patients do not respond adequately to first-line treatment and require second-line therapy with the addition of rituximab or cyclophosphamide [5]. Early initiation of therapy is a major prognostic factor, yet due to the lack of well-established diagnostic criteria, diagnosis is challenging. Furthermore, identifying NMDA receptor antibodies in blood or CSF may take days to weeks [5].

Evidence also suggests that treatment with immunosuppression alone may be insufficient to manage symptoms. Several cases reported in the psychiatry literature describe the use of electroconvulsive therapy (ECT) to manage dysautonomic, catatonic, and psychotic features that persist in children and adults long after immunosuppressive therapy is initiated [6, 7]. Here we report the case of a 16-year-old girl with confirmed anti-NMDA receptor encephalitis complicated by malignant catatonia that persisted despite aggressive immunosuppression and high-dose benzodiazepine (BZD) therapy. Her catatonia resolved only after ECT treatments.

## Case presentation

A previously healthy 16-year-old female with no contributing history presented with acute behavioral changes of emotional lability, lethargy, perseveration of speech, and opsoclonus-myoclonus. Initially, she was admitted to a psychiatric unit and received haloperidol and risperidone for agitation. Over the subsequent 4 days, she became less responsive, dysarthric, rigid, and developed fever as high as 103 ° F. In addition, she had a creatine kinase (CK) level of 913 U/L (9–185 U/L), which led to her admission to our pediatric intensive care unit for presumed neuroleptic malignant syndrome (NMS).

Initial physical exam showed a disoriented, confused, rigid adolescent girl with psychomotor slowing and blunted affect. She rapidly decompensated leading to respiratory compromise and urgent intubation. Dantrolene, lorazepam, and IV fluids were administered. Initial work-up showed normal complete blood count, complete metabolic panel, and thyroid panel. Anti-nuclear antibody, anti-double-stranded DNA, SSA, SSB, anti-Smith, ribosomal P antibody, thyroid antibodies, and complement levels were negative or normal; however, anti-ribonuclear protein (anti-RNP) was mildly elevated at 5 AI (normal < 1.0 AI). Serum immunoglobulins (Ig) IgG1 and IgG3 were raised. Cerebrospinal fluid (CSF) analysis revealed mild pleocytosis with 7 white blood cells (91% lymphocytes), normal protein, and mildly elevated oligo-clonal bands (4 bands). CSF meningitis and encephalitis PCR panels were negative for multiple bacterial and viral antigens, including HSV-1, VZV, WNV, and Cryptococcus. Computerized tomography with contrast of the chest, abdomen, and pelvis, as well as a pelvic ultrasound revealed no evidence of tumor. Her brain magnetic resonance imaging (MRI) with/without gadolinium was normal. A 24-h electroencephalogram displayed abnormal background slowing.

**Fig. 1:**
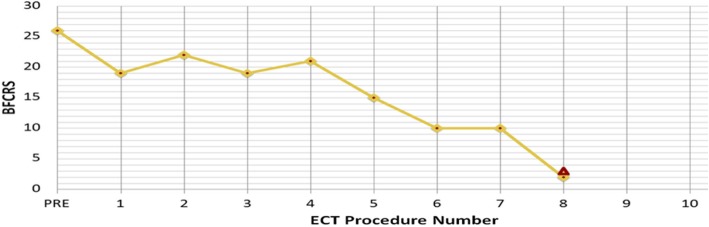
Bush Francis Catatonia Rating Scale (BFCRS) changes over ECT course. As shown, the patient’s BFCRS improved from 27 to 2 over 8 cycles of ECT. Mild improvement was noted over the first 4 treatments; however more significant improvement occurred between the 4th and 8th cycles

**Fig. 2:**
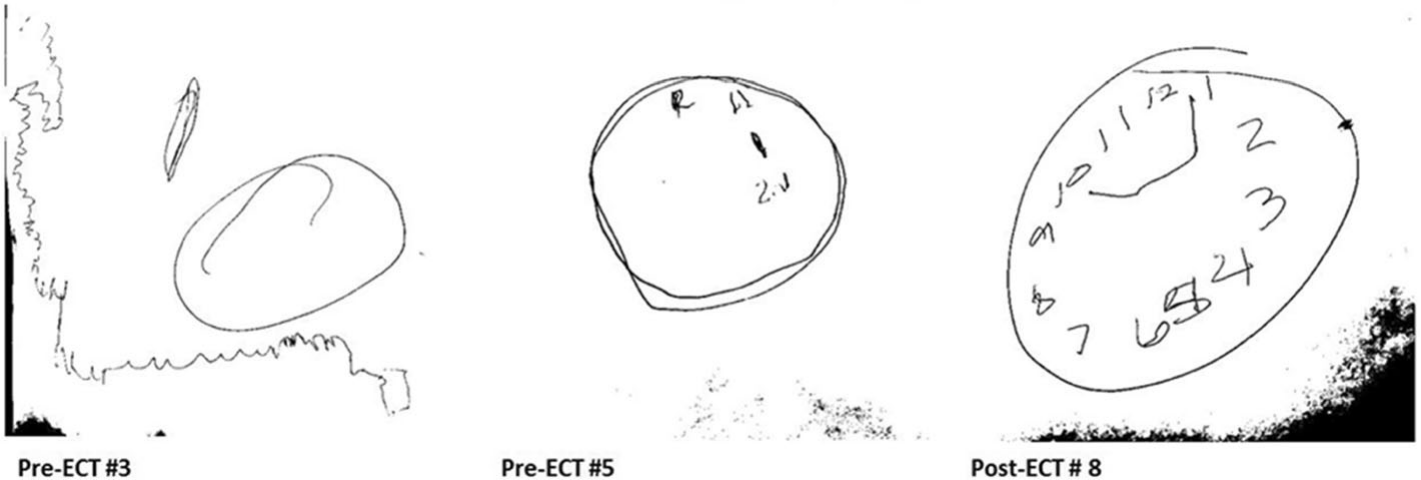
Clock Drawing Test (CDT). The patient’s mental status during ECT treatment, as evaluated by the CDT, showed marked improvement in visuospatial, motoric and cognitive functioning. Pre-ECT #3: CDT showed severe perseveration and conceptual deficits resulting in an inability to make any reasonable representation of a clock. Pre-ECT #5: CDT showed moderate visuospatial planning with disorganization and some perseveration (after drawing the contour, the patient set the numbers in wrong locations). Post-ECT #8: CDT showed a much improved visual representation of a clock with appropriate numbering

Early in her course, the NMS symptoms improved and she was extubated successfully. Observed generalized tonic-clonic seizures led to initiating levetiracetam. CSF results and her behavioral changes at presentation were suspicious for autoimmune encephalitis, therefore 5 days of methylprednisolone (dose 1 g/day) followed by 30 mg Q 12 h and PLEX (one cycle every other day for a total of 5 cycles) were begun empirically. Despite initial treatment, she remained emotionally labile, mute, intermittently agitated, rigid, and restless consistent with catatonia. Her catatonia remained refractory despite trials of lorazepam up to 18 mg/day and zolpidem up to 40 mg/day. On hospital day 9, serum and CSF samples confirmed anti-NMDA receptor autoimmune encephalitis. First-line treatment was intensified with IVIG (70 g every 2 weeks for 5 doses, then monthly for 3 extra doses). A rituximab course (1000 mg biweekly for 2 doses) was started as second-line immunosuppression with a second course of 1 g methylprednisolone daily for 3 days. In spite of these treatments, episodes of agitation, rigidity, sleep disturbance, mutism, facial twitching, drooling, and autonomic instability (heart rate 130–170; blood pressure 132–158/58–102) persisted leading to the diagnosis of malignant catatonia. Her Bush-Francis Catatonia Rating Scale (BFCRS) was 27 (normal is 0). Based on the recommendation of two independent psychiatrists and with family consent, ECT was begun to treat the malignant catatonia. ECT began on day 14 of her hospitalization with bitemporal electrode placement. A total of eight ECT treatments were given thrice weekly. Use of maximal device energies and the addition of flumazenil to reverse BZDs were necessary to achieve adequate seizure durations. Her heart rate and blood pressure improved after the fourth ECT and normalized after the seventh. Over the course of her ECT treatments, her BFCRS improved from 27 to 2 (Fig. 1). We also evaluated her mental status using the Clock Drawing Test (CDT) prior to ECT #3, ECT #5, and the day after ECT #8 (Fig. 2), showing marked improvement in visuospatial, motoric, and cognitive functioning. The patient’s mother noted after the course of ECT that the patient displayed more of her typical personality and mannerisms. Despite the improvement in physical strength, she continued to be mute at the termination of treatment. She was discharged home on melatonin 10 mg each night, levetiracetam, tapering doses of prednisone and lorazepam, and additional IVIG treatments as described above. She received intensive out-patient rehabilitation including speech, physical and occupational therapy. At her clinic visit two weeks post-hospital discharge, she was slow to respond verbally but had had obvious clinical improvement with the ability to talk in complete sentences and respond appropriately. Her verbal abilities and communication skills appeared normal at her 6-week post-ECT clinic appointment.

## Discussion

Since its discovery in 2007, anti-NMDA receptor encephalitis is being recognized more frequently, especially in adolescents and young adults [3]. As demonstrated by this case, the presentation of this autoimmune encephalitis may closely mimic primary psychiatric syndromes, but the disease process is unresponsive, or possibly exacerbated by psychotropic interventions.

Diagnosing anti-NMDA receptor encephalitis is often challenging given the wide range of clinical presentation, and may be further complicated by its overlap of symptoms, brain MRI changes, CSF findings with other entities affecting the brain. The differential diagnosis includes viral infections (e.g. HSV-1), other auto-immune processes (e.g. acute demyelinating encephalomyelitis or Hashimoto’s encephalitis), primary central nervous system vasculitis, or other described encephalitides. Even though the diagnosis can be made by identifying antibodies in immune-mediated encephalitis, the diagnosis may be delayed by weeks to months. One retrospective study found the median time from presentation to diagnosis to be 47 days for anti-NMDA receptor encephalitis, and even longer in other auto-immune encephalitides [8]. Delay in immune suppressive therapy is associated with adverse outcomes [9, 10]. Conventional clinical neurological assessment and standard diagnostic tests (MRI, EEG, and CSF studies) are reasonable approaches to decide early treatment of probable anti-NMDA receptor encephalitis since antibody testing or response to immunosuppression are late diagnostic criteria [11].

Immune suppressive therapies improve the prognosis of anti-NMDA receptor encephalitis. First-line treatment, including high-dose corticosteroids combined with either IVIG or PLEX or both, results in improvement of one-half of patients at 3–4 weeks. Second-line therapy (rituximab and/or cyclophosphamide) is efficacious in many patients with persistent symptoms after failing first-line treatment [5, 12, 13]. The choice and escalation of immune suppressive therapies depend on the severity of clinical symptoms and clinical response. In our case, early intense first-line treatment (high dose corticosteroids and PLEX) was started due to the severe clinical condition. Ten days later, after antibody confirmation of anti-NMDA receptor encephalitis and the failure to improve on first-line treatment, rituximab and IVIG were added. Even with the prompt initiation of first and second-line immunosuppression, the patient continued to suffer from malignant catatonia which required initiation of anti-catatonic treatments.

Malignant catatonia is a severe and life-threatening state associated with anti-NMDA receptor encephalitis that is often missed due to an under-appreciation of the condition and inadequate assessment skills [14]. Acute agitation and disorientation in our patient, along with autonomic instability prompted investigation for catatonia using the BFCRS, a standardized 23-item scale used to grade the severity of symptoms. It is the gold-standard for assessing the presence and severity of catatonia [15]. As in this case, it is often used to track response over time to anti-catatonic treatments.

Catatonia is a neuropsychiatric state with evidence of hypo-active gamma-aminobutyric acid-ergic (GABAergic) function. This is the primary target of initial treatment for all forms catatonia, which is high-dose BZD, usually lorazepam [16]. If this treatment fails, a course of ECT should be considered [17]. ECT is an effective and relatively safe treatment, including in children and adolescents [14], with transient cognitive side effects as the most concerning risk [18]. However, cases of severe catatonia treated with ECT actually show an improvement in cognition as their catatonia symptoms decrease in response to this intervention, as demonstrated in our patient, with improvement in her CDT [19]. Enhancement of GABAergic function is one of the known mechanisms of ECT that likely contributes to the anti-catatonic properties [20]. ECT treatment is supported by a recent systematic literature review which found complete (60%) or partial (33%) neurologic recovery in 30 cases of anti-NMDA receptor encephalitis complicated by severe psychiatric symptoms, primarily catatonia [21]. 

Our patient continued to manifest malignant catatonia in the face of prompt, aggressive immunotherapy and treatment with BZDs. Once ECT began, though, she had a robust response. Over eight emergent ECT treatments, her BFCRS score decreased to almost normal, and she displayed significant improvement in cognitive function as measured by the CDT. By 6-weeks post-ECT treatment, she had fluent verbal communication skills. After failing to respond to aggressive immune suppressive therapies and BZDs, our patient had a rapid improvement and complete resolution of her malignant catatonia after receiving ECT. This treatment was well-tolerated.

## Conclusion

Malignant catatonia is a severe and life-threatening neuropsychiatric complication of anti-NMDA receptor encephalitis that usually requires targeted treatment in addition to immune suppressive therapies. ECT was an effective and well-tolerated treatment of malignant catatonia in our patient and its use should be considered in children and adolescents with anti-NMDA receptor encephalitis whose catatonia does not respond to immunosuppression and benzodiazepines.

## Abbreviations

anti-RNP: Anti-ribonuclear protein
BFCRS: Bush-Francis Catatonia Rating Scale
BZD: Benzodiazepine
CDT: Clock Drawing Test
CK: Creatine kinase
CSF: Cerebrospinal fluid
ECT: Electroconvulsive therapy
EEG: Electroencephalogram
GABAergic: Gamma-aminobutyric acid-ergic
HSV-1: Herpes simplex virus-1
Ig: Serum immunoglobulins
IVIG: Intravenous immunoglobulin
MRI: Magnetic resonance imaging
NMDA: N-methyl-D-aspartate
NMS: Neuroleptic malignant syndrome
PLEX: Plasma exchange
VZV: Varicella-zoster virus
WNV: West Nile virus
